# The relationship between endometrial cancer and urinary incontinence: systematic review

**DOI:** 10.3389/fgwh.2026.1898627

**Published:** 2026-10-02

**Authors:** Feda Mohammed Ali Al Saleh, Khalil Ibrahim Bograin, Amjad Saud Alaqil, Nourah Mashari Alqadri, Aesha Abdulaziz Alamer, Kayan Munahi Alotaibi, Alwala’a Saud Alaqeel, Lubna Abdullatif Alnajim, Jomana Hussain Alali, Dhiyaa Abdullah Almusaylim, Majd Khalid Al Dhailan

**Affiliations:** 1 Eastern Health Cluster, Rural Health Network, Central Division, PHC Safwa, Safwa, Eastern, Saudi Arabia; 2 Alahas PHC Center, Al-Ahsa, Eastern, Saudi Arabia; 3 King Faisal University College of Medicine, Al Ahsa, Saudi Arabia

**Keywords:** cancer survivors, endometrial neoplasms, pelvic floor disorders, stress urinary incontinence, urge incontinence, urinary incontinence, uterine cancer

## Abstract

**Background:**

The most common gynecological cancer in industrialized countries is endometrial cancer (EC), whose prevalence is growing and is associated with metabolic syndrome and obesity. Women's quality of life is negatively impacted by the common and upsetting disorder known as urinary incontinence (UI), particularly in postmenopausal and post-surgical populations. Despite plausible biological and iatrogenic connections between EC and UI—including shared risk factors such as obesity and the impact of hysterectomy on pelvic anatomy—the relationship remains inadequately synthesized in the literature.

**Objective:**

The purpose of this systematic review is to summarize the data about the connection between endometrial cancer and incontinence, looking at prevalence, potential etiological mechanisms, and implications for clinical care.

**Methods:**

A comprehensive search for English-language studies published in the last ten years was done using PubMed/MEDLINE, Scopus, Web of Science, and the Cochrane Library. MeSH phrases and keywords associated with urinary incontinence and endometrial cancer were used in the search. Two reviewers used Rayyan software to independently pick studies, extract data, and evaluate quality. Because the studies were so different from one another, a narrative synthesis technique was used.

**Results:**

Six studies comprising 955 patients and 85 healthcare professionals met inclusion criteria. The collective evidence reveals that UI is a highly prevalent yet often undermanaged consequence of EC and its treatments. Findings demonstrate that lymph node dissection may contribute to urinary symptoms, though not reaching statistical significance. Radiotherapy exhibits paradoxical effects—potentially improving stress UI while contributing to broader pelvic floor dysfunction through radiation-induced fibrosis and measurable alterations in pelvic floor muscle properties. Obesity and age, rather than radiation exposure consistently, become important risk factors on their own.

**Conclusion:**

Due to common risk factors, direct effects of the disease, and damage from therapy, urinary incontinence is a common and clinically important side effect of endometrial cancer and its therapies. As the number of EC survivors keeps rising, addressing UI must become a priority in comprehensive survivorship care. Future efforts should focus on developing validated screening tools, establishing evidence-based management guidelines, and implementing integrated care models.

## Introduction

In industrialized countries, endometrial carcinoma (EC) is the most common gynecological cancer, and its incidence has been growing gradually in tandem with global increases in obesity and metabolic syndrome (1, 2). According to the International Agency for Research on Cancer (IARC) Global Cancer Observatory (GLOBOCAN 2022), cancers of the corpus uteri accounted for 420,368 new cases and 97,723 deaths worldwide in 2022, with a global age-standardized incidence rate of 8.4 per 100,000 population and an age-standardized mortality rate of 1.7 per 100,000. Endometrial cancer constitutes the majority of cancers arising in the corpus uteri and represents an important and increasing women's health concern (3). The disease primarily affects postmenopausal women, and its etiology is closely linked to prolonged, unopposed estrogen exposure. While advancements in early detection and treatment, particularly surgery, have led to favorable survival rates for early-stage disease, the long-term sequelae affecting survivors' quality of life are increasingly recognized as a critical component of comprehensive oncological care (4).

Urinary incontinence (UI) is one of the most common and upsetting disorders affecting women's quality of life, especially in the postmenopausal and post-surgical populations. Stress urinary incontinence (SUI), urge urinary incontinence (UUI), and mixed urinary incontinence (MUI) are the three main subtypes of urinary incontinence (UI), which jointly affect a significant percentage of adult women and whose incidence increases with age. UI is defined as the involuntary leakage of urin (5). The societal, psychological, and economic burdens of UI are profound, often leading to social isolation, depression, and significant healthcare expenditures (6). The pathophysiological underpinnings of UI are multifactorial, involving complex interactions between pelvic floor musculature, neuromuscular integrity, connective tissue support, and urethral function.

Clinical research on the relationship between pelvic floor dysfunction and gynaecological cancer is well-established but intricate. Urinary symptoms have been investigated in relation to cervical and ovarian cancers, frequently in the context of drastic surgical procedures or radiation treatments that directly jeopardise pelvic anatomy and innervation (7). In the present literature, the precise relationship between endometrial cancer and urinary incontinence is still largely unexplored and inadequately summarised. There are a number of iatrogenic and biological pathways that are worth investigating. First, there is a strong correlation between the two conditions and common risk factors, particularly obesity. Due to long-term increases in intra-abdominal pressure, Obesity is a major cause of type I endometrial cancer through estrogen-driven processes and a substantial independent risk factor for stress urinary incontinence (8). Second, the recognized primary therapy for endometrial cancer is complete hysterectomy, which is often coupled with bilateral salpingo-oophorectomy. Urinary incontinence may be caused or exacerbated by this procedure because it changes the pelvic anatomy and may interfere with the pelvic nerve plexus and the bladder and urethra's supporting structures (9).

Despite these compelling connections, the existing body of evidence is fragmented across observational studies of varying designs and quality. To date, no comprehensive synthesis has been conducted to critically evaluate the strength, consistency, and nature of the association between endometrial cancer and urinary incontinence. The purpose of this systematic review is to methodically compile the data about the connection between endometrial cancer and incontinence.

## Methodology

### Study design

To guarantee clear and comprehensive reporting of procedures and results, this systematic review was carried out in compliance with the Preferred Reporting Items for Systematic Reviews and Meta-Analyses (PRISMA) criteria (10). The study topic, search strategy, inclusion and exclusion criteria, data extraction techniques, and quality evaluation methodologies were all specified in the *a priori*-developed review procedure.

### Search strategy and information sources

A systematic literature search was conducted in PubMed/MEDLINE, Scopus, Web of Science, and the Cochrane Library. The search strategy combined controlled vocabulary (MeSH terms) and free-text keywords related to endometrial cancer and urinary incontinence, including “endometrial neoplasms,” “endometrial cancer,” “uterine cancer,” “urinary incontinence,” 'stress urinary incontinence,' “pelvic floor disorders,” and “pelvic floor dysfunction.” Boolean operators (AND and OR) were used to combine the search terms. The reference lists of included studies and relevant review articles were also manually screened to identify potentially eligible studies that might not have been retrieved through the electronic database searches.

The literature search was conducted from January 2016 to May 2026. Two reviewers independently screened titles and abstracts and subsequently assessed potentially eligible full-text articles according to the predefined eligibility criteria. Disagreements were resolved through discussion and, when necessary, consultation with a third reviewer. Rayyan was used to manage references, remove duplicates, and facilitate the screening process (11). This systematic review was not prospectively registered in PROSPERO.

### Review question and PICOS framework

The review question was structured according to the PICOS framework:

- Population (P): Adult women diagnosed with endometrial cancer.
- Intervention/Exposure (I): Endometrial cancer and/or cancer-directed treatment, including surgery, lymph-node dissection, radiotherapy, and other relevant treatment modalities.
- Comparator (C): A non-exposed group, alternative treatment group, or other comparison group when available in the individual studies.
- Outcomes (O): Urinary incontinence, including stress, urge, or mixed urinary incontinence, and related urinary or pelvic-floor outcomes.
- Study design (S): Observational and interventional studies, including prospective and retrospective cohort studies, cross-sectional studies, and experimental/interventional designs.

### Study selection process and Rayyan utilization

All identified records from the database searches were exported to Rayyan, A web-based program for systematic reviews that makes it easier for people to screen and choose papers together (12). Rayyan was utilized throughout the screening process to organize references, remove duplicate records, and enable efficient blinded screening by reviewers. There were two separate stages to the research selection procedure. Two reviewers independently reviewed the titles and abstracts of all retrieved records against the predetermined eligibility criteria in the first phase. Any differences between reviewers were settled by discussion or, if required, contact with a third reviewer. Records that blatantly failed to fulfill the inclusion criteria were eliminated. In order to verify eligibility, two reviewers independently evaluated the entire texts of possibly eligible papers in the second round. Studies were excluded at the full-text stage for verified reasons. Rayyan tracked and recorded the whole selection process, guaranteeing the review methodology's repeatability and openness. The study selection process is illustrated in Figure 1.

**Figure 1:**
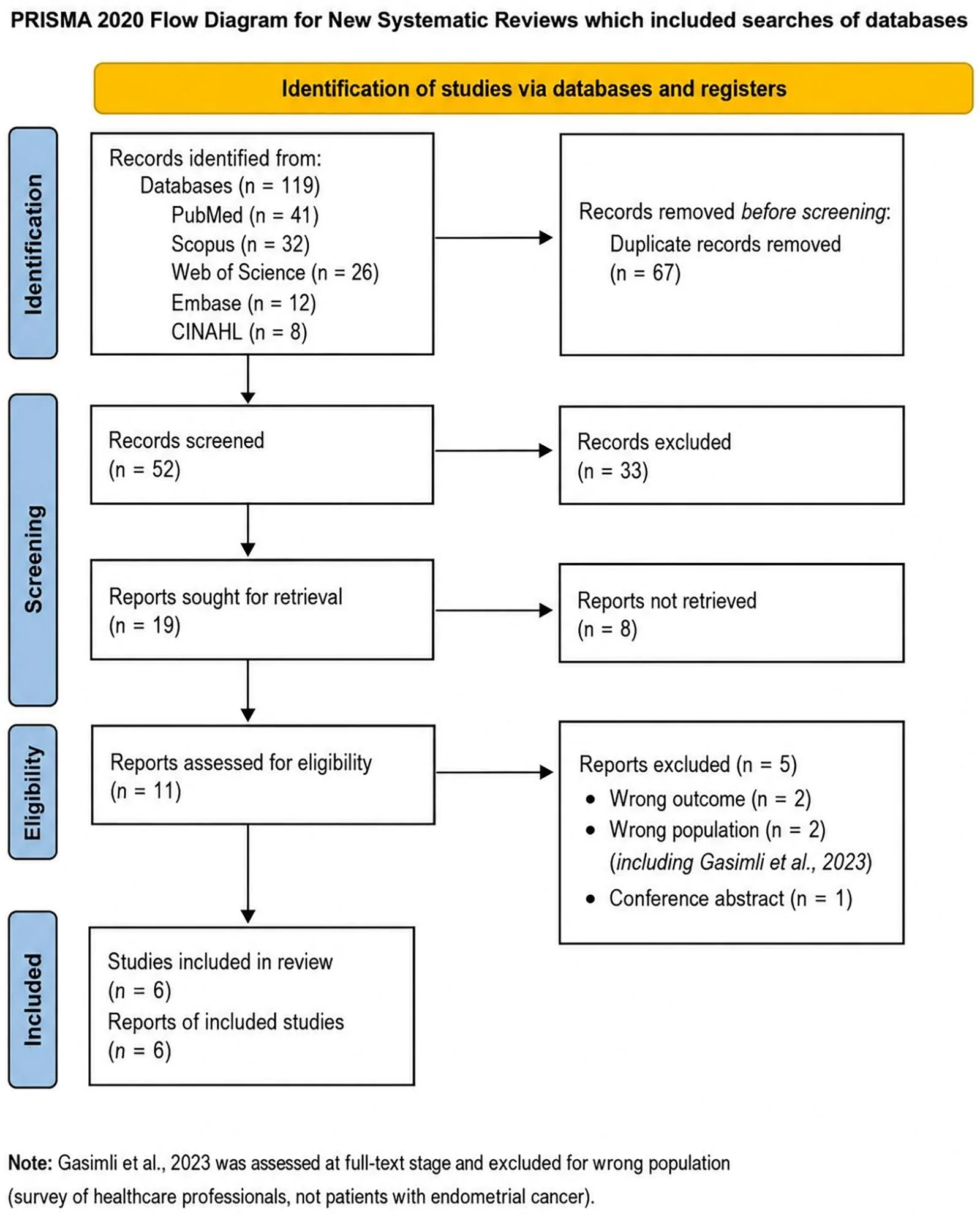
PRISMA Flow Diagram of Study Selection Process.

### Eligibility criteria and study selection

Studies that satisfied the following requirements were deemed qualified for inclusion: (1) comprised adult females with a diagnosis of endometrial intraepithelial neoplasia or endometrial cancer; (2) examined urinary incontinence as a primary or secondary outcome measure; (3) utilized innovative study designs, such as cross-sectional, interventional, prospective, or retrospective cohort studies; (4) utilized validated instruments or objective measures to assess urinary incontinence; and (5) were printed in English-language, peer-reviewed journals.

### Eligibility criteria

Studies were eligible if they included adult women with endometrial cancer and evaluated urinary incontinence or related urinary or pelvic-floor outcomes. Studies including women with endometrial intraepithelial neoplasia (EIN) together with women with endometrial cancer were also eligible when the study population and outcomes were directly relevant to endometrial cancer and its treatment. Studies focusing exclusively on EIN without any endometrial cancer population were excluded.

Studies were excluded if they were case reports, case series with fewer than 10 participants, conference abstracts, editorials, commentaries, narrative reviews, or systematic reviews. Studies focusing exclusively on other gynecologic malignancies were excluded unless separate data for women with endometrial cancer were available. Studies focusing exclusively on endometrial intraepithelial neoplasia without endometrial cancer were also excluded.

### Data extraction process

To systematically gather pertinent data from every included study, a consistent data extraction form was created beforehand. Data from all qualifying papers was separately retrieved by two reviewers, and disagreements were settled by consensus. Included in the retrieved data were: (1) study attributes: sample size, study design, place of origin, first author, and year of publication; (2) characteristics of participants: description of the population, average age, average BMI, stage of cancer, and type of treatment received; (3) urinary incontinence assessment: measurement tools used, timing of assessment, and specific incontinence outcomes reported; (4) key findings: main results related to urinary incontinence prevalence, severity, and associated factors; and (5) research findings as stated by the writers. An attempt was made to get in touch with the respective authors of studies with incomplete or ambiguous data, and when this was not possible, missing data were recorded as “not mentioned” (NM) in the data extraction tables.

### Quality and risk of bias assessment

Using instruments suitable for each research design, the included studies' methodological quality and bias risk were evaluated. The National Institutes of Health (NIH) Quality Assessment Tool for Cohort and Cross-Sectional Studies was used for both prospective and retrospective cohort studies. The clarity of the research topic, the study population definition, the participation rate, the consistency of exposure and outcome assessment, blinding, loss to follow-up, and confounding variable correction are among the important methodological components that are assessed by this instrument. For cross-sectional studies, the same NIH tool was utilized with appropriate modifications, whereas a modified version of the AXIS tool, which was created especially for the critical evaluation of cross-sectional research, was used to evaluate the cross-sectional survey of medical professionals. The Risk of Bias Assessment Tool for Non-randomized Studies (RoBANS) was used for the single-case experimental design investigation. A quality grade of “good,” “fair,” or “poor” was given to each study according to the evaluation of its particular criteria. Two reviewers separately completed the quality evaluation, and disputes were settled by discussion.

### Data synthesis and analysis

A meta-analysis was not acceptable nor practical because of the significant variability across included studies with regard to design, demographics, treatments, outcome measures, and follow-up lengths. Rather, the findings were compiled and integrated using a narrative synthesis technique. Key themes that emerged from the included research served as the framework for the narrative synthesis: (1) the impact of surgical treatment on urinary incontinence, including the role of lymph node dissection and concomitant incontinence procedures; (2) the effect of radiotherapy on pelvic floor function and incontinence; (3) the pathophysiological mechanisms underlying incontinence, including objective measures of pelvic floor muscle properties; (4) evidence for conservative management strategies and rehabilitation; and (5) clinical practice patterns and barriers to incontinence care. Findings were presented descriptively in tables and text, with careful attention to the strength and consistency of evidence across studies.

## Results

PRISMA flow diagram illustrates the systematic study selection process, beginning with the identification of 119 records through database searching. Following the removal of 67 duplicate records, 52 unique records proceeded to title and abstract screening, where 33 records were excluded for not meeting the eligibility criteria. The remaining 19 reports were sought for retrieval. Eight reports could not be retrieved through the available retrieval methods, and the remaining 11 full-text reports were assessed for eligibility. During the eligibility assessment phase, Five reports were excluded after full-text assessment: two because of wrong outcome, two because of wrong population, and one because it was a conference abstract. Ultimately, 6 studies met all inclusion criteria and were included in the final systematic review synthesis.

Table 1 provides a comprehensive overview of the demographic and clinical characteristics of the Six studies, the geographical distribution of the research is diverse, encompassing studies from Turkey (13), the United States (14–16), Canada (17, 18). The study designs are varied, including prospective cohort studies (14), Cohort studies conducted in the past (15, 16), studies that are cross-sectional (17), and a single-case experimental design (18), reflecting the different levels of evidence available on this topic. The sample sizes range considerably from a small, in-depth interventional study of only 8 participants (18) to a large prospective cohort of 532 patients (14). Mean ages across the patient cohorts are generally in the early 60s, and mean BMI values are consistently in the overweight to obese range (27.5 to 36.0 kg/m^2^), which aligns with the known risk profile for endometrial cancer. The cancer stages reported include early-stage disease (EIN and FIGO Stage I) (14, 15) as well as mixed stages including more advanced cases (16), while others did not specify the stage (13, 17, 18). In accordance with standard care paradigms for endometrial cancer, patients are generally treated surgically (hysterectomy with or without lymph node dissection), frequently in conjunction with adjuvant radiation.

**Table 1:** Demographic and clinical characteristics of included studies.

Study (First Author, Year) [Ref.]	Study Design	Sample Size (N)	Population Description	Mean Age (years)	Cancer Stage	Treatment Received
Erkilinç, S., et al. (2025) (13)	Prospective Cohort Study	78	EC patients post-surgery, with or without adjuvant RT	59.2 (RT group), 60.6 (non-RT group)	NM	Total hysterectomy + bilateral salpingo-oophorectomy ± RT
Robison, K., et al. (2023) (14)	Prospective Cohort Study	532	Patients with EIN or early-stage EC and SUI symptoms	61.0 (concomitant surgery), 62.0 (cancer surgery only)	EIN or Early-stage EC	Cancer surgery (hysterectomy) ± concomitant SUI surgery
Lipetskaia, L., et al. (2019) (15)	Retrospective Cohort Study	74	Patients with FIGO Stage I EC who underwent robotic-assisted TLH	NM	FIGO Stage I	Robotic-assisted TLH ± LND
Segal, S., et al. (2017) (16)	Retrospective Cohort Study	149	EC survivors, with or without exposure to RT	64.1 (No RT), 63.1 (VBT only), 63.8 (WPRT ± VBT)	All stages (Mix of I, II, III)	Surgery ± VBT or WPRT
Bernard, S., et al. (2017) (17)	Cross-sectional Study	29	Women with UI after hysterectomy and RT for EC (*n* = 11) vs. women with hysterectomy only and no UI (*n* = 18)	62.9 (EC + UI group), 59.4 (Control group)	NM	Hysterectomy + RT (for affected group)
Bernard, S., et al. (2021) (18)	Single-Case Experimental Design	8	EC survivors with UI, at least 1-year post-primary treatment	61.9	NM	Surgery + RT (62.5%), or Surgery only (37.5%)

Table 2 synthesizes the core outcomes and findings related to urinary incontinence from each included study, providing a direct link to the primary objective of this systematic review. The aims of the studies are diverse but collectively explore the relationship from multiple angles: investigating specific treatments like radiotherapy (13, 16) or lymph node dissection (15) as risk factors for UI, comparing surgical approaches for UI management (14), examining the underlying physiological mechanisms (17), testing rehabilitative interventions (18). A range of validated assessment tools were employed to measure UI and its impact, including condition-specific questionnaires such as the Incontinence Severity Index (ISI) (13), Incontinence Impact Questionnaire (IIQ-7) (12, 14), Urogenital Distress Inventory (UDI-6) (15), International Consultation on Incontinence Questionnaires (ICIQ) (17, 18), as well as the Pelvic Floor Distress Inventory (PFDI-20) (13), as well as objective measures like the pad test (18) and intravaginal dynamometry (17, 18). The key findings reveal a complex picture: while radiotherapy may improve stress UI symptoms in some patients (13), it is not consistently identified as an independent risk factor for UI (16) and is associated with alterations in pelvic floor muscle function (17). Importantly, UI in this population appears to be amenable to intervention, as an in-home rehabilitation program demonstrated significant reductions in UI severity (18). The main conclusions consistently emphasize the need for greater attention to UI as a critical aspect of survivorship care, with calls for structured education, multidisciplinary cooperation and the creation of clinical recommendations (13–18).

**Table 2:** Outcome measures and key findings related to urinary incontinence.

Study (First Author, Year) [Ref.]	UI Assessment Tool(s)	Prevalence/rate	95% CI	*P* value	Key Findings on UI	Effect Estimates
Erkilinç, S., et al. (2025) (13)	Turkish-validated ISI, IIQ-7, PFDI-20	NR	NR	NR	RT group had lower SUI incidence and ISI scores but higher PFDI-20 scores. Non-RT group had higher SUI and ISI scores.	Turkish-validated ISI, IIQ-7, PFDI-20 RT group had lower SUI incidence and ISI scores but higher PFDI-20 scores; non-RT group had higher SUI and ISI scores. NR for exact effect estimate in the current manuscript.
Robison, K., et al. (2023) (14)	FACT-En, Questionnaires for urinary symptom severity	NR	−1.3 to 3.6	NR	Concomitant SUI surgery did not result in a clinically significant improvement in cancer-related QOL compared to cancer surgery alone. QOL improved in both groups post-surgery.	FACT-En; urinary symptom severity questionnaires Adjusted median change: 1.2 points (95% CI −1.3 to 3.6). Concomitant surgery: 150 vs. 72.5 mL blood loss, *p* < 0.001; operative time 185.5 vs. 152 min, *p* < 0.001.
Lipetskaia, L., et al. (2019) (15)	UDI-6, IIQ-7	NR	NR	0.104	Patients who underwent LND reported higher, but not statistically significant, scores on UDI-6 and IIQ-7 compared to those without LND.	UDI-6, IIQ-7 UDI-6: 30.0 vs. 20.7, *p* = 0.104; difference was not statistically significant.
Segal, S., et al. (2017) (16)	Validated questionnaires for UI (specific names NM)	48% vs. 58%	Age: 1.02–1.10 BMI: 1.02–1.11	0.47 (for RT comparison)	Rates of UI were similar between women exposed and not exposed to RT. Age and BMI, but not RT, were significant risk factors for UI on multivariable analysis.	Validated UI questionnaires UI: 48% vs. 58%, *p* = 0.47; age: aOR 1.06 (95% CI 1.02–1.10); BMI: aOR 1.07 (95% CI 1.02–1.11).
Bernard, S., et al. (2017) (17)	ICIQ-UI SF, ICIQ-OAB, ICIQ-B	NR	NR	NR	The EC + UI group showed significant reductions in maximal vaginal length, PFM maximum force, rate of force development, and number of rapid contractions.	ICIQ-UI SF, ICIQ-OAB, ICIQ-B EC + UI group showed significant reductions in maximal vaginal length, PFM maximum force, rate of force development, and rapid contractions.
Bernard, S., et al. (2021) (18)	Pad test (primary), ICIQ-UI SF, 3-day bladder diary, intravaginal dynamometry	87.5% had reduced UI severity	NR	NR	The program resulted in a reduction in UI severity for 87.5% of participants. Significant improvements were seen in some PFM dynamometric tests.	Pad test, ICIQ-UI SF, 3-day bladder diary, intravaginal dynamometry UI severity was reduced in 87.5% of participants. RTE = 0.30

BMI, Body Mass Index; EC, Endometrial Cancer; EIN, Intraepithelial Neoplasia of the Endometrium; FACT-En, Functional Assessment of Cancer Therapy-Endometrial; FIGO, International Federation of Gynecology and Obstetrics; ICIQ-B, International Consultation on Incontinence Questionnaire - Bowels; ICIQ-OAB, International Consultation on Incontinence Questionnaire - Overactive Bladder; ICIQ-UI SF, International Consultation on Incontinence Questionnaire - Urinary Incontinence Short Form; IIQ-7, Incontinence Impact Questionnaire-7; ISI, Incontinence Severity Index; LND, Lymph Node Dissection; N/A, Not Applicable; NM, Not Mentioned; PFDI-20, Pelvic Floor Distress Inventory-20; PFM, Pelvic Floor Muscles; PFMT, Pelvic Floor Muscle Training; QOL, Quality of Life; RT, Radiotherapy; SUI, Stress Urinary Incontinence; TLH, Total Laparoscopic Hysterectomy; UDI-6, Urogenital Distress Inventory-6; UI, Urinary Incontinence; VBT, Vaginal Brachytherapy; WPRT, Whole Pelvic Radiation Therapy.

Table 3 provides an evaluation of the risk of bias for each of the six included studies, which is an essential part of any systematic review to assess the quality and internal validity of the evidence compiled. The evaluation was carried out using instruments especially chosen for each research type, as explained below: the NIH Quality Assessment Tool for cohort and cross-sectional studies, the RoBANS tool for the single-case experimental design, and a modified version of the AXIS tool for the cross-sectional survey. The evaluations show that the included studies' overall quality ranges from mediocre to good. The research of prospective cohorts (13, 14) were rated as “Good” due to their clear objectives, well-defined populations, use of validated instruments, and consideration of key confounding variables. The retrospective cohort studies (15, 16) received “Fair” ratings, primarily due to the inherent limitations of their design, such as potential selection bias and reliance on medical records, although they still used appropriate measurement tools. The cross-sectional study by Bernard et al. (17) was rated “Good” for its clear methodology and direct comparison of relevant groups. The single-case experimental design (18) was also rated “Good,” demonstrating a robust methodology for this type of study.

**Table 3:** Risk of bias assessment for included studies.

Study (First Author, Year) [Ref.]	Study Design	Risk of Bias Tool Used	Key Criteria Assessed	Overall Rating	Domain-Level Judgement
Erkilinç, S., et al. (2025) (13)	Prospective Cohort Study	NIH Quality Assessment Tool for Cohort and Cross-Sectional Studies	Clear research question, defined study population, >50% participation rate, consistent use of validated measures (ISI, IIQ-7, PFDI-20), justification of sample size, exposure measured before outcome, adequate timeframe, various exposure levels examined, use of validated outcome measures, blinding of outcome assessors, loss to follow-up <20%, and measurement and adjustment of key confounding variables.	Good	Low risk across most assessed domains, including study population, participation, exposure measurement, outcome measurement, follow-up, and confounding. Some concerns regarding outcome-assessor blinding.
Robison, K., et al. (2023) (14)	Prospective Cohort Study	NIH Quality Assessment Tool for Cohort and Cross-Sectional Studies	Clear research question, well-defined study population, greater than 50% participation rate, and regular use of verified metrics (FACT-En), Justification of sample size, exposure quantified before outcome, adequate timeframe, examination of various exposure levels, use of validated outcome measures, blinding of outcome assessors, loss to follow-up <20%, and measurement and adjustment of important confounding factors (using adjusted median regression).	Good	Low risk across most assessed domains, including study population, participation, outcome measurement, exposure assessment, and adjustment for confounding. Some concerns regarding blinding of outcome assessors.
Lipetskaia, L., et al. (2019) (15)	Retrospective Cohort Study	NIH Quality Assessment Tool for Cohort and Cross-Sectional Studies	A well-defined research issue, a study population, a participation rate more than 50%, and the regular use of validated measures (UDI-6, IIQ-7) There is no explanation for the sample size, exposure is evaluated before the result, there is not enough time to explore various exposure levels, validated outcome measures are not used, outcome assessor blinding is not disclosed, loss to follow-up is not relevant, and important confounding factors are not quantified or corrected.	Fair	Low risk for research question, study population, participation, and use of validated measures. Some concerns regarding sample-size justification, exposure assessment/timeframe, outcome-assessor blinding, and control of confounding.
Segal, S., et al. (2017) (16)	Retrospective Cohort Study	NIH Quality Assessment Tool for Cohort and Cross-Sectional Studies	Clear research question, defined study population, unreported participation rate (mailed survey), consistent use of validated measures, unjustified sample size, exposure assessed before outcome, adequate timeframe, various exposure levels examined (VBT, WPRT), use of validated outcome measures, unapplied blinding of outcome assessors (survey), unapplied loss to follow-up, and measurement and adjustment of key confounding variables (age, BMI in multivariable analysis) are all important factors.	Fair	Low risk for the research question, study population, validated measures, and temporal assessment of exposure. Some concerns regarding participation rate, sample-size justification, and control of potential confounding.
Bernard, S., et al. (2017) (17)	Cross-sectional Study	NIH Quality Assessment Tool for Cohort and Cross-Sectional Studies	Clear research question, defined study population, >50% participation rate, consistent use of validated measures (ICIQ, dynamometry), no justification for sample size (small, exploratory), no exposure, no adequate timeframe, examined exposure levels, use of validated outcome measures, no blinding of outcome assessors, no loss to follow-up, and no measurement or adjustment of key confounding variables.	Good	Low risk for research question, study population, participation, and outcome measurement. Some concerns due to the small exploratory sample, lack of exposure assessment/timeframe, absence of assessor blinding, and limited assessment of confounding.
Bernard, S., et al. (2021) (18)	Single-Case Experimental Design	RoBANS (Risk of Bias Assessment Tool for Non-randomized Studies)	Selection of participants (low risk), confounding variables (low risk, as participants served as their own controls), measurement of exposure (low risk, objective pad test and validated questionnaires), blinding of outcome assessments (low risk), incomplete outcome data (low risk), selective outcome reporting (low risk).	Good	Low risk for participant selection, confounding, exposure measurement, and outcome measurement based on objective pad testing and validated questionnaires. Some concerns related to the small sample size and single-case experimental design.

## Discussion

Evidence reveals that urinary incontinence is a highly prevalent yet often undermanaged consequence of endometrial cancer and its treatments, with significant implications for quality of life in survivorship.

Numerous studies have examined the effects of surgical intervention on urinary function in individuals with endometrial cancer, with differing findings. Lipetskaia together with associates (15) discovered that lower urinary tract symptoms were not substantially impacted by lymph node dissection during robotic-assisted laparoscopic hysterectomy; the lymphadenectomy group's mean UDI-6 scores were 30.0, while the control group's were 20.7 (*p* = 0.104). Obesity and urinary incontinence have a particularly pertinent link, as Segal and colleagues (16) demonstrated that BMI (aOR 1.07, 95% CI 1.02–1.11) and age (aOR 1.06, 95% CI 1.02–1.10) were significant independent risk factors for urinary incontinence in endometrial cancer survivors, while radiation exposure showed no significant association.

The role of adjuvant radiotherapy in pelvic floor dysfunction has been a subject of considerable debate in the literature. The prospective cohort study by Erkilinç and colleagues (13) revealed a paradoxical effect of radiotherapy: while the non-radiotherapy group had higher rates of stress urinary incontinence and higher Incontinence Severity Index scores, The overall Pelvic Floor Pelvic Floor Dysfunction Index overall score and its component values were much higher for the radiation group (13). The objective pelvic-floor measurements reported by Bernard et al. (17) provide evidence of pelvic-floor functional impairment in women with urinary incontinence after endometrial cancer treatment. Conversely, Segal and colleagues (16) found no association between radiation exposure and urinary incontinence in their retrospective cohort of 149 endometrial cancer survivors, with incontinence rates of 48% in women exposed to radiotherapy compared to 58% in those not exposed (*p* = 0.47). The discrepancy between these findings may reflect differences in study design, patient populations, and radiation characteristics (13, 16). Additionally, the Segal study included a heterogeneous group of patients receiving vaginal brachytherapy alone or whole-pelvic radiation with or without brachytherapy, while the Erkilinç study did not specify the type of radiotherapy delivered (13, 16). These differences highlight the need for more standardized reporting of radiation exposure in future studies.

Robison and associates (14) compared patients who chose concurrent cancer and stress urinary incontinence surgery (*n* = 110) to those who underwent cancer surgery alone (*n* = 422) in a large multicenter prospective cohort study of 532 patients with endometrial intraepithelial neoplasia or early-stage endometrial cancer and stress urinary incontinence symptoms. From preoperative to postoperative periods, both groups' quality of life, as determined by the Functional Assessment of Cancer Therapy-Endometrial (FACT-En), improved significantly. After controlling for covariates, the concomitant surgery group's median change scores were 1.2 points higher (95% CI −1.3 to 3.6) (14). However, the study did demonstrate that concomitant surgery was associated with longer time to surgery (22 vs. 16 days, *p* < 0.001), greater estimated blood loss (150 mL vs. 72.5 mL, *p* < 0.001), and longer operative time (185.5 min vs. 152 min, *p* < 0.001) (14).

Recent evidence also highlights the importance of accurate preoperative risk stratification in patients with endometrial intraepithelial neoplasia (EIN), given the possibility of concurrent endometrial carcinoma and the potential need for more extensive surgical staging. Çaltek et al. (2025) evaluated preoperative predictors of concurrent endometrial carcinoma in patients undergoing hysterectomy for EIN and emphasized the value of identifying patients at higher risk who may require lymph node dissection. Although this study did not directly evaluate urinary incontinence, its findings are clinically relevant to survivorship because the extent of surgical staging may influence postoperative pelvic-floor and urinary outcomes (19).

Sexual health is another important component of survivorship that may complement urinary and pelvic-floor outcomes. In a 2025 cross-sectional study, Güner et al. evaluated sexual function in women with endometrial cancer and sexual satisfaction among their partners after surgery alone or surgery followed by adjuvant brachytherapy. Among 69 women, no statistically significant differences were observed between treatment groups in Female Sexual Function Index scores or partner sexual satisfaction after adjustment for age. Nevertheless, sexual function was generally impaired among endometrial cancer survivors, highlighting the importance of addressing sexual health and partner-related outcomes as part of comprehensive survivorship care (20).

Although limited, evidence for conservative management of urinary incontinence in endometrial cancer survivors is emerging. Bernard and associates (18) conducted a single-case experimental design research on eight endometrial cancer survivors who took part in a pelvic floor muscle training program at home with assistance from mobile technology. According to the results, 87.5% of individuals experienced less severe urinary incontinence, and the pad test showed a significant relative treatment effect of moderate size (RTE: 0.30) (18). The rapid contraction and endurance dynamometric tests also showed significant modest relative treatment effects (18). These findings indicate a potential benefit of mobile technology-assisted pelvic-floor muscle training for urinary incontinence in endometrial cancer survivors; however, the small sample size limits the generalizability of these findings (18). The included studies were heterogeneous in terms of study design, patient populations, treatment characteristics, urinary incontinence assessment tools, and outcome definitions. Therefore, findings were synthesized narratively, and direct comparisons or quantitative pooling were considered inappropriate where substantial clinical and methodological heterogeneity was present.

### Limitations

When evaluating the results, it is important to take into account the many limitations of this systematic study. First, there is a significant amount of design variability across the included research, populations, and outcome measures, which precluded meta-analysis and limits the ability to draw quantitative conclusions. The studies ranged from prospective cohort designs to retrospective analyses and cross-sectional surveys, each with inherent methodological strengths and weaknesses. Second, the sample sizes across studies were generally modest, with the smallest interventional study including only eight participants (18), limiting statistical power and generalizability. The retrospective cohort studies (15, 16) were particularly susceptible to selection bias and confounding, as they relied on medical records and could not fully account for differences in baseline characteristics between comparison groups. Third, it is challenging to separate the effects of endometrial cancer and its therapies from age-related changes in pelvic floor function since most studies lacked a non-cancer control group. Just one study (17) included a comparison group of women with hysterectomy for benign indications without urinary incontinence, providing some insight into the specific effects of cancer treatment beyond surgery alone. Fourth, the assessment tools for urinary incontinence varied across studies, including the Incontinence Severity Index, Urogenital Distress Inventory-6, Incontinence Impact Questionnaire-7, International Consultation on Incontinence Questionnaire, and Pelvic Floor Distress Inventory-20, among others. While all these instruments are validated, their different scoring systems and domains of assessment complicate cross-study comparisons. Fifth, the duration of follow-up varied considerably, from three months post-treatment (13) to studies including patients up to ten years after diagnosis (16). The natural history of urinary incontinence after endometrial cancer treatment may evolve over time, and short-term follow-up may not capture late-emerging symptoms. Sixth, studies with favorable outcomes had a higher chance of being published than those with null results, raising concerns about publication bias. The inclusion of studies with conflicting findings regarding the effect of radiotherapy on urinary incontinence (13, 16) partially mitigates this concern but does not eliminate it. Last but not least, linguistic bias may have been introduced by excluding papers written in languages other than English, thereby leaving out pertinent research from non-English speaking nations where patient demographics and practice patterns may differ. Potential retrieval bias should also be acknowledged, as eight reports could not be retrieved for full-text assessment. Although predefined eligibility criteria were applied consistently, the inability to access these reports may have introduced retrieval bias and potentially affected the completeness of the evidence base. A pooled prevalence estimate of urinary incontinence could not be calculated because of substantial heterogeneity among the included studies in study populations, assessment instruments, definitions of urinary incontinence, and timing of outcome assessment. Consequently, prevalence estimates were presented descriptively rather than quantitatively pooled.

## Conclusion

A considerable percentage of survivors experience urinary incontinence, a common and clinically important side effect of endometrial cancer and its therapies that impairs quality of life. Urinary incontinence and endometrial cancer have a complex association that reflects common risk factors such as advanced age and obesity, the direct impact of the disease on pelvic floor tissues, and damage after radiation and surgery. The evidence regarding radiotherapy's specific contribution remains mixed, with studies showing both improvement in stress incontinence symptoms and worsening of overall pelvic floor dysfunction, highlighting the need for larger prospective investigations with standardized outcome measures. As the population of endometrial cancer survivors continues to grow, addressing urinary incontinence must become a priority in comprehensive survivorship care to optimize quality of life and functional outcomes for women after cancer treatment. Future efforts should focus on developing validated screening tools, establishing evidence-based management guidelines, and implementing integrated care models that ensure all survivors have access to appropriate assessment and intervention services.

## Data Availability

The original contributions presented in the study are included in the article/Supplementary Material, further inquiries can be directed to the corresponding author.
